# Newer, Older, and Alternative Agents for the Eradication of *Helicobacter pylori* Infection: A Narrative Review

**DOI:** 10.3390/antibiotics12060946

**Published:** 2023-05-23

**Authors:** György Miklós Buzás, Péter Birinyi

**Affiliations:** 1 Ferencváros Health Centre, Gastroenterology, Mester utca 45, 1095 Budapest, Hungary; 2 Medoc Health Centre, Gastroenterology, Lehel út 8, 1137 Budapest, Hungary; 3 Department of Pharmacodynamics, Faculty of Pharmacy, Semmelweis University, Szentkirályi utca 46, 1086 Budapest, Hungary; pbirinyi@gmail.com

**Keywords:** bismuth compounds, capsule therapy, carbonic anhydrase inhibitors, fluoroquinolones, *Helicobacter pylori*, macrolides, potassium-competitive acid blockers, probiotics, proton pump inhibitors, registry

## Abstract

Although discovered 40 years ago, *Helicobacter pylori* infection is still raising diagnostic and therapeutic problems today. The infection is currently managed based on statements in several guidelines, but implementing them in practice is a long process. Increasing antibiotic resistance and weak compliance of the patients limit the efficacy of eradication regimens, leaving much room for improvement. Third-generation proton pump inhibitors have added little to the results of the first two generations. Potassium-competitive acid blockers have a stronger and longer inhibitory action of acid secretion, increasing the intragastric pH. They obtained superior results in eradication when compared to proton pump inhibitors. Instead of innovative antibiotics, derivatives of existing antimicrobials were developed; some new fluoroquinolones and nitazoxanide seem promising in practice, but they are not recommended by the guidelines. Carbonic anhydrase inhibitors have both anti-secretory and bactericidal effects, and some researchers are expecting their revival in the treatment of infection. Capsules containing components of the eradication regimens have obtained excellent results, but are of limited availability. Probiotics, if containing bacteria with anti-*Helicobacter pylori* activity, may be useful, increasing the rates of eradication and lowering the prevalence and severity of the side effects.

## 1. Introduction

Discovered 40 years ago, *Helicobacter pylori* (*H. pylori*) infection continues to raise diagnostic and therapeutic problems. Thousands of randomised controlled trials (RCTs) have been performed, and dozens of guidelines have been published, but the ideal treatment for eradicating the infection is still elusive. The reasons for this are the increasing prevalence of antibiotic resistance rates across nearly the whole world and all compounds, coupled with weak compliance/adherence to the regimens administered [1]. Additionally, the lack of approval by authorities and the limited availability of some drugs in some countries contributes to the problems encountered by current therapies [2]. Antimicrobial resistance testing is limited to dedicated centres and is far from being generally applied, even in developed countries. Antimicrobial stewardship was recently designed for the responsible and appropriate use of antibiotics, and an improved efficacy of eradication regimens is expected [3]. Opinion leaders and experts have formulated guidelines for the management of *H. pylori* infection, but have often drawn different conclusions [4,5,6], which were later reconciled [7]. In Europe, the Maastricht VI/Florence report is the most recent guideline [8]. In fact, neither of the statistical methods, including network meta-analysis, have resulted in universally valuable conclusions [9,10].

As shown by the European Registry on the management of *Helicobacter pylori* (Hp-EuReg), which includes 21,533 patients, the results of eradication therapies in different European regions are heterogenous, suboptimal, and at variance with the current recommendations [11], leaving much room for improvement (i.e., do not use ineffective standard triple therapies again, prescribe therapy for 10–14 days, do not repeat the same antibiotics after a failure, always check the eradication result, and verify the compliance of patients with the treatment) [12]. Shifting the treatment from the 7-day triple regimens given initially to the quadruple therapies of 10–14 days is a long-lasting process, which needs further dissemination of knowledge and education [11]. This is an excellent territory of study for registries.

Given the inconsistencies in the current eradication results, the aim of our narrative review is to present some older and newer drugs/regimens which can be used as alternatives to the existing standard treatments. Data on the first and second PPI generations and “classical antibiotics”, such as amoxicillin (A), clarithromycin (C), metronidazole (M), and the worldwide resistance profiles of these antimicrobials are not presented here; these are covered extensively by several recent excellent reviews, and duplicating this information would be superfluous.

## 2. New Generation of Proton Pump Inhibitors (PPIs)

The efficacy of PPIs has been proven unequivocally, but some unmet needs remain, which has contributed to a decrease in their eradication performance. The effects of most antibiotics are pH-dependent, so new or modified PPIs have been synthetised in the hope for an increased intragastric pH for longer periods, more profound acid inhibition than with the earlier compounds, and less interindividual variability.

Ilaprazole (IL) is a substituted benzimidazole with a longer plasma half-life than the first two generations of PPIs. It achieves a higher intragastric pH (>6) for longer periods than esomeprazole (ESO) [13]. IL is approved for use and marketed only in China and South Korea. There is a paucity of eradication studies with IL (Table 1) [14,15]. Since no head-to-head comparative studies with classical PPIs have been conducted, no meta-analysis is possible. Judging based on the eradication rates, the pharmacologic advantages bring no additional benefit over earlier PPIs.

Dexlansoprazole (DL) is an R-enantiomer of lansoprazole (LS), which was originally synthetised in Japan and has a longer and stronger acid inhibitory action than the parent compound, with a 3–5-fold area under the plasma concentration–time curve. It has a dual-release (DR) formulation, hitting peak concentrations one and four hours after ingestion [16]. In a first pilot study, Graham et al. found that high-dose DL with an A-based dual therapy was ineffective [17]. A head-to-head comparison with ESO in triple and quadruple regimens revealed acceptable rates of eradication. Although DL is one of the most popular PPIs prescribed in the United States, most studies have been performed in the Far Eastern countries [18,19,20,21] (Table 1).

**Table 1:** Third generation proton pump inhibitor effects on *H. pylori* eradication: results of randomised studies.

Ref.	Year	Country	Study Treatment (mg/Day)	Control Treatment (mg/Day)	Duration (Days)	No. of Patients	Eradication (PP)	Conclusion
[14]	2022	China	IL 2 × 5 + A 2 × 1000 + F 2 × 100, BiG 2 × 220 mg	(a) IL 2 × 5 + A 2 × 1000, F 2 × 100, BiG 2 × 220 mg (b) Il 2 × 5 + A 4 × 750	14/10/14	133/136/142	94.7/87.5/93.0%	The efficacy of 14-day double therapy with IL+A is similar to that of quadruple therapies for 10 and 14 days, with better compliance and lower cost.
[15]	2022	China	IL 2 × 5 + D 2 × 100 + F 2 × 100 + Bi 2 × 220 mg	IL 2 × 5 + A 2 × 1000 + F 2 × 100 + Bi 2 × 220	14/14	92/92	92.9/91.8%	A- and D-based quadruple regimens given for 14 days are of the same efficacy, D being a useful alternative in the case of penicillin allergy.
[18]	2016	China	DL 1 × 60 mg+A 2 × 1000 mg + C 2 × 500 mg	R 2 × 20 mg + A 2 × 1000 mg + C 2 × 500 mg	7/7	90/87	85.1/81.2%	Both regimens achieved suboptimal results. DL has a lower cost than R.
[19]	2015	Thailand	DL 2 × 60 + LEV 500 + C 1000, BiS 2 × 1048	DL 2 × 60, LEV 500, C 1000, BiS 2 × 1048	14/7	48/42	98%/85.7%	The 14-day DL-containing quadruple regimen provided a higher eradication rate than the same regimen given for 7 days.
[20]	2019	Taiwan	DL 1 × 60 + A 2 × 1000 mg + C 2 × 500 mg	(a) ESO 2 × 40 mg + A 2 × 1000 mg + C 2 × 500 mg (b) ESO 4 × 40 mg + A 2 × 1000 mg + C 2 × 500 mg	7/7/7	63/75/77	93.7/94.7/89.6%	High-dose DL triple therapy was non-inferior to ESO-based 7-day therapies, achieving acceptable results.
[21]	2019	Taiwan	DL-MR 4 × 60 mg, A 2 × 1000, C 2 × 500 + M 2 × 500 mg	L 2 × 30 mg + A 2 × 1000 mg + C 2 × 500 mg + M 2 × 500 mg	7/7	96/98	90.6/90.1%	DL-MR-based 7-day quadruple therapy was equivalent to L-based concomitant therapy.

Abbreviations: A: amoxicillin, BiG: bismuth glycyrrhizinate, BiS: bismuth subsalicylayte; C: clarithromycine, D: doxycycline, DL: dexlansoprazole, DL-MR: dual-release dexlansoprazole, F: furazolidone, ESO: esomeprazole, IL: ilaprazole, LEV: levofloxacin, LS: lansoprazole, M: metronidazole. R: rabeprazole, T: tetracycline. In column 7, the first figure is the number of patients in the study group, followed by the number of the patients in the control group(s). In column 8, the first figure is the eradication rate in the study group, and the second and third figures are the eradication rates in the control group(s).

Tenatoprazole (TP) is a prodrug of omeprazole (O) with a longer plasma residence time, obtaining a prolonged and greater acid suppression than ESO. Its S-enantiomer was more efficient than ESO in suppressing 24 h of diurnal and nocturnal acidity, but its development was not continued [22].

In spite of their pharmacologic advantages, the new PPIs have not obtained superior results compared to the older ones in the eradication of *H. pylori* infection.

## 3. Potassium-Competitive Acid Blockers (P-CAB)

Although PPIs represent major progress in the management of acid-related diseases and *H. pylori* eradication, at least when compared to H_2_-histamine receptor inhibitors, the unmet needs of both patients and doctors must be improved by other types of acid inhibitors [23,24]. P-CABs were developed in the 1980s. The first compound that inhibited the K^+^-site of the parietal cell H^+^/K^+^-ATP-ase was SCH28080, but it was later withdrawn because of hepatotoxicity. Other P-CABs were not superior to ESO and also caused drug-induced liver changes. The Korean drug revaprazan, introduced in 2005, proved to be equivalent to PPIs. Vonoprazan fumarate (VPZ) was marketed in Japan in 2015 and approved later by the Food and Drug Administration and European Medicine Agency (2016). It has several pharmacologic and clinical advantages over PPIs (Table 2), being a stronger, more stable, and long-acting acid inhibitor than the PPIs currently used [1,2,23,24,25,26].

Several open and randomised control trials compared the efficacy of VPZ-based regimens with those of PPI-based treatments, resulting in three meta-analyses and systematic reviews [27,28,29,30]. (Table 3). In another meta-analysis, the VPZ proved to be superior to the PPI-based therapies as second-line regimens (odds ratio of 1.51, 95% confidence interval: 1.27–1.81, *p* < 0.001) [31].

Recently, the results of a randomised European and United States study were published, showing the superiority of VPZ-based regimens over LS-based triple therapy. The eradication rate of VPZ-based triple therapy was 80.8% on an ITT base, compared to 58.5% with LS-based therapy (difference of +12.3%, 95% CI 5.7–18.8%, *p* < 0.001). The PP eradication rates were 85.7% and 70.0% (difference of +15.7%, 955% CI: 8.9–22.5%, *p* < 0.001). VPZ-based dual therapy achieved 77.2% eradication versus 68.5% with LS-based triple therapy (difference of +8.7%, 95% CI 1.9–15.4, *p* < 0.13). On a PP basis, the eradication rates were 81.1% and 70.0% (difference of 11.1, 95% CI 3.9–18.2%, *p* = 0.03). In C-resistant strains, the P-CAB-based regimen was successful in 65% of the patients compared to 31.9% with the LS triple regimen (difference of 33.9, 95% CI 17.7–48.1, *p* < 0.001). The PP eradication rates were 67.2% and 29,0%, respectively (difference of 38.2%, 95% CI 20.5–53.4, *p* < 0.001). Adverse effects were reported in 34.1% and 29.9% of the cases with VPZ-based triple and dual therapies and 34.5% in the PPI-based triple therapy group [32].

Both the Far Eastern and the European–North American results proved the superiority of VPZ-based therapies over PPI-based ones. The incidence of the side effects was comparable. While even a recent network meta-analysis showed that VPZ-based regimens were superior to PPI-based ones (9); accepting Graham’s grading, they are not yet ideal (i.e., constantly obtaining >95% success) [33]. Perhaps VPZ given as a bismuth-based or concomitant quadruple 14-day regimen will reach this magic rate at some point.

Tegoprazan (TGZ) is a new P-CAB with the same acid-inhibitory effect as VPZ. In a randomised Korean study, TGZ 50 mg-based triple therapy (A 1000 mg/day + C 500 mg/day) was not inferior to an LS-based triple therapy, and its effect was not affected by the CYP2C19 status, but it did not overcome the C resistance. The eradication rates were low, with 62.8% vs. 60.5% on an ITT basis, and 69.3% vs. 67.3% on a PP basis [34]. In another Korean study, TGZ-based A+C triple therapy for 10 days achieved 76.4% on an ITT basis, while 10 days of concomitant therapy containing TGZ+A+M+C obtained 85.9% (*p* < 0.001). The PP-based values were 84.5% and 91.1%, respectively (*p* < 0.001). The adherence was similar between the therapies [35].

Time and experience will determine if P-CABs can overcome PPIs for the eradication of *H. pylori* infection; initially, there were high hopes for each regimen.

## 4. New Antibiotics

Soon after the introduction of antibiotics in the management of *H. pylori* infection, it became clear that with most complex regimens, resistance to antibiotics is driven by chromosomal mutations, the alteration of efflux systems, and biofilm formation [36]. The resistance is historically dynamic and variable across countries, geographic regions, the use of antibiotics, and local strain profile, as well as the patients’ compliance and adherence. *H. pylori* was listed as a high-priority microorganism, deserving of the development of new antibiotics, behind those of carbapenem-resistant *Acinetobacteria baumanni*, *Pseudomonas aeruginosa*, and *Enterobacteriaceae*; these multi-drug resistant microbes were considered a critical research priority [37]. Most of the antibiotics used for eradication are products of the prime time of pharmacological research after the Second World War, often intercalated with serendipities. As a consequence, instead of innovative antibiotics, rather new compounds of the existing groups were synthetised; a few of them have been experimented with in the eradication of infection, as shown below.

### 4.1. Penicillin Derivatives

With its low resistance rates, A is the mainstay of eradication regimens except for in penicillin-allergic patients, for whom alternative regimens have been proposed [36,38]. It has been known for 20 years that beta-lactamase inhibitors (clavulanic acid, sulbactam, and tazobactam) have antibacterial action [39]. Eradication rates can be augmented by clavulanic acid [40]. Second-generation cephalosporin cefuroxime does not cause penicillin allergy, but its use is rather limited and not recommended by the guidelines [4,5,6,7,8]. In a recent Chinese study, ESO, cefuroxime, LEV, and bismuth given for 14 days obtained rates of 85.5% (95% CI: 79.6–90.8%) on an ITT basis and 90.1% (95% CI: 85.2–94.4%) on a PP basis, with a resistance rate of 4% for the cephalosporin [41]. Other new penicillin containing beta-lactamase inhibitors (meropenem–vaborbactam, imipenem–relebactam) have not been tried either in vitro or in vivo against *H. pylori*. Hence, this class of drugs has not added much to the eradication rates, maintaining the stable role of A as the first-line medicine.

### 4.2. Macrolides

After the success and decline of C as a main component of eradication regimens, second-generation (miokamycin, rokitamycin, and tilmicosin) and third-generation macrolides (telithromycin, cethromycin, and solithromycin, referred to as ketolides) were synthetised. All are derivatives of the parent molecule erythromycin, isolated in 1952 [42]. Regardless of their generation, all macrolides bind to the ribosomal subunit of the bacteria, with the macrolactone ring oriented to the ribosomal tunnel. All compounds interact via a hydrogen bond between the lactone ring and the nucleotide A2058 of the 23S rRNA. They also interact with the bacterial efflux pumps. Azithromycin and roxithromycin given for 3, 5, or 7 days have produced variable results in the past and raised similar resistance problems [42].

Telithromycin had an MIC_50_ value of 0.12 µg/mL and an MIC_90_ value of 0.15 µg/mL (higher than that of A and C) against *H. pylori*, but its development was stopped because of hepatotoxicity [43]. Solithromycin, with its MIC_50_ value of 0.12–8 µg/mL, was equivalent to or less active than LEV, but retained its activity in macrolide-resistant respiratory infections. [44]. There have been no studies on the use of other new macrolides as anti-*H. pylori* agents.

### 4.3. Fluoroquinolones

Fluoroquinolones (FQs) have been used for over 50 years. Their first two representatives, ofloxacin and ciprofloxacin, were obvious, but the rapid occurrence of high resistance rates prevented their more extensive use [45]. LEV, an enantiomer of ciprofloxacin, is recommended as the first-line and rescue treatment by most of the recent guidelines [4,5,6,7,8], although the rates of resistance are steadily increasing as in the case of macrolides [36]. Since then, several fourth-line FQs have been synthetised: sitafloxacin (STF), gatifloxacin (GTF), delafloxacin (DLF), garenoxacin (GRX), finafloxacin (FNF), gemifloxacin, and levonadifloxacin. STF, GRX, and FNF are stronger than LEV against *H. pylori*, and some of them have overcome resistance to previous quinolones [46].

Most experience was gathered with STF, a Japanese drug synthetised in 2008. It has better in vitro activity against *H. pylori* than LEV. STF was introduced in Japan mainly as a third-line PPI- or VPZ-based regimen, in association with A, M, or even rifabutin. In a recent meta-analysis, 7 days of PPI+STF+A given as a third-line treatment obtained a mean eradication rate of 70.1% (95% CI: 59.0–79.2), while VPZ and the same combination of antibiotics achieved 88.9% (95% CI 74.5–95.4%). The PPI-STF+M combination given for 7–14 days obtained rates of 76.4–91.1%, but a direct comparison with PPI or VPZ regimens containing A was not performed. The authors considered STF a good option for rescue eradication [47]. The side-effect profile was favourable.

GTF was introduced in 1999 against respiratory tract infections. GTF given for 7 days with A and rabeprazole was efficient as second-line therapy in 84.4% of cases (95% CT: 74–95%), but only in 63.6% of cases as a third-line regimen [48]. Sequential treatment with esomperazole+A followed by GTF for 12 days resulted in rates of 80% (95% CI:61–92%) on an ITT basis and 85.8% (95% CI: 67–95%) on a PP basis [49]. However, dysglycaemia and other side effects, such as dizziness and diarrhoea, restrained its use.

FNF is a novel FQ more active than LEV or moxifloxacin in an acidic environment (pH 5.0–7.0), but its activity is limited to strains with certain *gyr A* mutations [50].

Lastly, the large-spectrum DLF, produced since 2006, was found to have a much lower MIC than LEV against *H. pylori* (MIC_50_ 0.0106 vs. 0.125 and MIC_90_ 0.125 vs. 32 µg/mL, respectively). In both cases, the clinical studies are lacking [50,51].

Feared complications of FQ treatment include tendon ruptures, retinal detachment, and increased risk of aortic aneurysm or dissection. In a recent meta-analysis of nine studies, FQs increased the risk of these vascular complications by 69% (pooled risk ratio: RR = 1.69 (95% CI:10.8–2.64)), but only observational studies were analysed, with high heterogeneity and publication bias. Neither of the studies referred to *H. pylori* eradication and no case presentation is known. Clinically, one can hardly believe that 7–14 days of FQ treatment could change the aortic architecture [52]. Instead, during the decades of infection, *H. pylori* itself could promote the development of vascular complications (coronary heart disease, stroke, etc.) and even infect the existing aortic aneurysms [53].

In conclusion, the new FQs have not brought about any real progress in the management of *H. pylori* infection.

### 4.4. Tetracyclines

Tetracycline was synthetised in 1959 and it became a component of the first—then, PPI-free—regimen against *H. pylori*, as proposed in 1990 at the World Congress of Gastroenterology in Sydney [54]. It was frequently administered until the advent of one-week triple combinations consisting of a PPI+A+C, as proposed by the Maastricht I. consensus [55], which overshadowed its use until the increase in antibiotic resistance to penicillin and macrolides. Thus, the Maastricht/Florence VI consensus recommended a PPI+bismuth+T+M combination as the first-line treatment, regardless of C resistance [8]. T binds reversibly to the 30S subunit of bacterial ribosomal 16S-RNA, with a bacteriostatic and bactericidal effect by inhibiting protein synthesis. In contrast with most antimicrobials, resistance to T has been stable in recent decades, albeit with variations worldwide (4–10%) [36,56]. Although it obtains high rates of eradication, its use is limited by gastrointestinal and unpredictable side effects (liver damage, allergy, photosensitivity, asthma, and haemolytic anaemia). In spite of these, it was included in the three-in-one capsule, achieving favourable eradication rates (see below). In a small recent study, given with the P-CAB VPZ, T obtained 100% eradication as the first-line and 97% as the second-line treatment [57].

Doxycycline (D) (1967) is a first-generation analogue of T, with an enhanced stability and increased lipophilicity. As shown by a meta-analysis, its eradication efficacy in triple and quadruple therapy is similar to that of T [58].

Minocycline is a second-generation semi-synthetic tetracycline, produced since 1965. With almost complete bioavailability and increased lipophilicity, it has increased tissue penetration, which could be advantageous in accessing hidden sites of *H. pylori.* Unfortunately, it also crosses the blood–brain barrier, causing nausea, dizziness, and headache, as well as hyperpigmentation and gum and scleral discoloration [59]. Due to its anti-inflammatory effect, minocycline has been successfully tried in acne vulgaris, periodontal disease, rheumatoid arthritis, and neuroinflammatory and neurodegenerative diseases, as well as atherosclerosis. In the past 20 years, minocycline was tested in *H. pylori* treatment; the results were summarized in a recent systematic review showing that when given for 7–10 days as quadruple therapy, it achieved eradication rates of 95%, while the effect of triple therapies was only 70% [60]. Due to its side effects, however, the drug is not recommended by the guidelines as first- or second-line or rescue treatment (4–8). The Hp-EuReg data show that it is not used in European practice [61].

Third-generation Ts are tigecycline, omadacycline, eravacycline, and sarecycline, and all are derivatives of the parent compound tetracycline, discovered in 1953 [54]. Tigecycline and omadacycline exhibit potent in vitro activity against *H. pylori*, inhibiting bacterial protein synthesis. Both drugs inhibit the mutations of 16S rRNA that mediate resistance to tetracycline and have secondary binding sites, resulting in stronger antibacterial action. Oral administration has recently become available and has successfully been applied in *Clostridioides difficile*, *Acinetobacterium baumani*, and *Cutinebacterium acnes* infections, but the high price will probably hinder their use as anti-*H. pylori* drugs [62,63].

### 4.5. Nitazoxanide and Nitroimidazoles

Nitazoxanide (NTZ) is a thiazolidon with bactericidal properties similar to metronidazole, inhibiting the pyruvate oxidoreductase enzyme. It is largely used in Egypt against parasitic infections. NTZ is active against *H. pylori*, with an MIC of 0.25–8 µg/mL, and avoids nitroimidazole resistance [64]. After promising pilot studies and randomised studies, a meta-analysis and systematic review selected six RCTs and seven open studies including 1028 patients. Regimens containing NTZ obtained a pooled eradication rate of 86% (95% CT: 79–90%). The heterogeneity, however, was high (I^2^: 84%). Given as a second-line treatment, NTZ obtained a rate of 85% (95% CI: 69–94%), without heterogeneity. The highest rates were observed if NTZ was given with LEV, doxycycline, and a PPI; in this setting, rates of 92% (95% CI: 88–95%) were obtained. More importantly, a favourable safety profile was reported in the included studies [65]. Probably because of the paucity of large-sized RCTs, NTZ was not recommended for use in any of the recent guidelines [4,5,6,7,8].

Combinations of M with other chemicals have been prepared in the hope of avoiding resistance. A combination of linolenic acid with M provided an acid-resistant compound with stronger activity against *H. pylori* and lower MIC_90_ values than the individual components alone [66]. Conjugates of rifamycin with several nitroimidazoles (M, pretomanid, delamanid, and NTZ) resulted in active compounds against *H. pylori*, *Clostridioides difficile*, and *Staphylococcus aureus*, being dual-targeted synergistic chemicals, with the hope of inhibiting the growth of hidden bacteria using biofilms [67]. Human studies are lacking.

### 4.6. Nitrofurans: Furazolidone and Nitrofurantoin

Furazolidone (F) and nitrofurantoin (NF) are nitroheterocyclic and nitroaromatic compounds that were synthetised in the 1950s. These furan-based compounds are among the oldest antimicrobials. The susceptibility of *H. pylori* to nitrofurans has been known since 1986, with the MIC_90_ being 0.5 μg/mL for F and 1.0 μg/mL for N [68]. Resistance to F was detected earlier in 1.8–8% of *H. pylori* isolates, while NF resistance rates were under 1%, and this has not grown in recent years [36]. Although the mechanism of resistance to F and NF is not completely understood, it probably does not involve genes related to M resistance. F was used with success in China for treating peptic ulcers long before *H. pylori* discovery [69]. In spite of its limited availability, F is extensively used in Asia and South America. There are also some studies from the USA and Europe. Three meta-analyses have been performed, with somewhat contradictory results. In the first meta-analysis in 2007, PPI-based, F-containing triple therapies achieved a rate of 76.3%, bismuth-based triple regimens 84.5%, and quadruple regimens obtained eradication in 83.4% of the cases. Second-line schedules were efficient in 76.1% and third-line regimens in only 65.5% of the cases. The duration of treatment, but not the F dose, influenced the outcome. Side effects occurred in 34% of the cases with F and 28% of the controls [70]. In an Italian work-up from 2012, F-based first-line triple therapies obtained 75.7% on an ITT basis and 79.5% on a PP basis; a high F dose and longer duration of treatment achieved better results [71]. Both meta-analyses showed that F-based regimens obtained suboptimal results. Finally, in a Chinese study from 2018, only bismuth-containing quadruple regimens obtained acceptable results (eradication rate: 92.9%; 95% CI: 90.7–95.1%). The incidence of the total and severe side effects revealed no significant difference compared to the controls [72]. A recent systematic review revealed no increased risk of adverse effects during F treatment, even with a high dose (200 mg/day) [73]. Earlier, however, concerns were raised regarding the carcinogenetic effect of F in rodents [74]. Its main advantage is the low price. The drug is banned in the USA and in some European countries, while the Western guidelines do not recommend its use for eradication purposes, and, as shown by the Hp-EuReg, it is rarely used by European practitioners (11,38,61). However, the drug is considered underutilised in Iran [75].

NF was one of the first drugs used for eradicating *H. pylori*, but subsequent studies have revealed unacceptable results. Graham et al. obtained eradication with quadruple therapy (NF 3 × 100 mg, omeprazole 2 × 20 mg/day, bismuth subsalicylate 3 × 2 × 262 mg/day mg, T 3 × 500 mg/day) in only 70% (95% CI: 50.6–85.0%) of the cases. The treatment was more effective in M-sensitive (88%) than M-resistant cases (33%) [76]. Given as a third-line regimen after failed triple therapies, a combination of pantoprazol 2 × 40 mg + NF 3 × 100 mg + A 2 × 1000 mg + T 4 × 500 mg achieved rates of 61.9% (95% CI: 52.6–71.2%) on an ITT basis and 65.0% (95% CI: 56.2–77.2%) on a PP basis [77]. In spite of the inadequate results, NF was given in 0.7% of the cases in a recent Swedish study on 157,915 eradication episodes [78]. Interstitial pneumonitis and nephritis have been occasionally described as complications of NF treatment, but we never came across these in our practice.

## 5. Carbonic Anhydrase Inhibitors

Carbonic anhydrase (CA) was isolated in 1933. In 1943, it was observed that its main class of inhibitors—the sulphonamides—produce alkaline diuresis. Acetazolamide (AZ) was synthetised in 1952 as the first potent inhibitor of CA and started to be used clinically as a diuretic in 1954. Long before the discovery of *H. pylori*, AZ was used to inhibit gastric acid secretion in France and the USA. Later, Puşcaş et al. in Romania treated a large number of peptic ulcer patients with an original drug composed of AZ and a mixture of Na and K bicarbonate, citrate, and bismuth subnitrate, to compensate for electrolytic losses. He obtained high endoscopic healing rates (over 90%) for gastric and duodenal ulcers at the expense of an inconvenient side-effect profile, which meant the drug did not achieve widespread use even in its country of origin, not to mention abroad [79]. Ethoxzolamide (ETZ) led to similar results, with a more acceptable side-effect profile [80]. Moreover, the 2-year rate of recurrence after healing was very low without continuous maintenance treatment, suggesting other mechanisms of action besides the inhibition of acid secretion, due to human gastric CA inhibition [81]. In the *H. pylori* era, it was shown that *H. pylori* encodes for both α-CA and β-CA, located either in the cytoplasm (β-CA) or in the periplasmic space (α-CA). ETZ has antimicrobial activity against different strains of *H. pylori* (SS1, 26695), with MIC values of 0.3–0.5 mM, and a frequency of resistance at <5 × 10^−9^ [82]. The bactericidal activity of ETZ is concentration-dependent [83]. It seems, therefore, that ETZ is the first drug with both anti-secretory and antibiotic effects. As with most antibiotics, resistance against ETZ can be raised by complex nucleotide changes, targeting the genes involved in cell wall synthesis and the CA genes encoded by the bacterium. CA inhibitors are also active against other bacteria, such as *Salmonella enterica*, *Brucella suis*, and *Pseudomonas aeruginosa* [84]. It was suggested that CA plays a role in the adaptation of *H. pylori* to the acid environment and, moreover, it helps bacteria survive in biofilms. In addition to AZ and ETZ, many other substituted benzene sulphonamides have been synthetized, but none have entered the clinical scene, although some bacterial CAs were validated as anti-infective drug targets (85). Based on the chemical, experimental, and microbiological research data, it is envisaged once more that CA inhibitors could represent a new class of anti-ulcer and anti-*H. pylori* drugs. The association of CA inhibitors with probiotics lacking this enzyme could also be beneficial, improving the antibacterial effect of the former and stimulating the host immune system [85]. However, so far, no RTC investigating the in vivo effect of CA inhibitors on *H. pylori* eradication has been performed.

## 6. Capsule Therapy

Capsules have been used for more than two centuries for the manufacture of medicines, making them more suitable for oral intake. While the eradication of *H. pylori* is mandatory for all infected patients, there is still no therapy achieving a 100% eradication rate, as is common with other infectious diseases. In addition to the rates of antimicrobial resistance increasing worldwide, weak compliance with the more or less complicated regimens is the main cause of their decreasing efficacy. Bismuth compounds and T are often unavailable in many countries. The purpose of realising a three-in-one capsule was to avoid the inconvenience of taking too many tablets per day for relatively prolonged periods (10–14 days), thus improving the compliance and eradication rates.

Bismuth is given as a single agent in many capsule forms (salicylate, potassium citrate, pectin, etc.), and without other antibiotics; these were not taken into consideration here, although many studies have proven their efficacy [86].

The inclusion of bismuth subcitrate (120 mg), M (125 mg), and T (100 mg) in one capsule, administered in combination with omeprazole (20 mg), was proposed in 2000 and marketed under the name Pylera^®^ (Allergan Inc, Dublin). It was used in the first- and second-line and rescue therapy of *H. pylori* infection. In 2011, a European phase III trial showed that when administered for 10 days, the capsule treatment was superior to 7-day triple therapy containing C [87]. A meta-analysis from 2018, based on 30 studies and 6482 patients, showed eradication rates of 90% on an ITT basis (CI: 87–92%) in first-line, 89% (CI: 86–93%) in second-line, and 82% (CI: 78–87%) in rescue therapy [88]. It was also efficient in M- and C-resistant cases and in patients previously treated with macrolides. The efficacy was independent of the PPI dose and type. The side effects occurred in 43% of the cases, leading to a cessation of the treatment in 3% of the cases. Although the therapy consists of the intake of 3 × 3 capsules/day, this did not limit compliance. The drug was approved for use by the FDA and the EMA. Its availability is, however, limited in some countries, and its price could be prohibitive in low-income regions.

There has been no head-to-head comparison of capsule therapy with bismuth-based quadruple treatment with the same composition (BQT). However, a meta-analysis from 2013 showed that BQT for 10 days achieved an eradication rate of 78.2%, which is 12% lower than the rate obtained with capsule therapy [89]. The reasons for this difference are unclear (different pharmacokinetics or compliance).

The success of the capsule treatment raised interest in other capsule preparations. The purpose was either to reduce the costs of or include novel substances. In Hungary, 120 mg of bismuth citrate and 375 mg of M were included in a gelatine capsule and given q.i.d with a PPI and T for 7, 10, and 14 days as second-line treatment. The eradication rate was 90% after 7 days, 95.4 % after 10 days, and 100% after 14 days. In spite of the promising results, the capsule can be purchased only as magistral preparation [90,91].

Similarly, Italian authors prepared galenic capsules containing 20 mg rabeprazole, 200 mg bismuth salicylate, 500 mg T, and 500 mg M, obtaining a success rate of 92.9% on an ITT basis and 95.1% on a PP basis, with mild side effects. The replacement of colloidal bismuth subcitrate with homemade bismuth salicylate offers the advantage of *H. pylori* flagellin synthesis inhibition, reducing its motility. Bismuth salicylate is an easily available pharmaceutic compound in regions where other drugs (De-Nol^®^, Pylera^®^) are not accessible [92].

In another study in Egypt, 250 mg LEV was prepared in the form of mini-tablets and incorporated into capsules. It was verified for pharmacologic properties (thickness, hardness, friability, and content). The in vitro release of the drug was similar to that of a commercial product. The authors considered the new formulation to be promising for the eradication of *H. pylori*, although no microbiological or clinical testing was performed [93].

Capsules have also been used in alternative medicine. In China, the volatile oil Jinghua Weikang obtained from the medical plant *Chenopodium ambrosinoides* was mixed with dimethyl sulfoxide and encapsulated (JWC). It was shown that JWC killed *H. pylori* in standard cultures and those in biofilms as well, which were 10–1000 times more resistant to antibiotics. The mechanisms of action are the inhibition of bacterial cell division and that of ATP-binding cassette (ABC) transporters, the latter playing a main role in the function of the efflux pump families of *H. pylori*. In vitro, JWC significantly decreased the minimum inhibitory concentration (MIC) of M, reversing the resistance to this antimicrobial [94]. There is a lack of human studies so far.

As a physical method, blue light was shown to kill cultured *H. pylori* at a wavelength of 405 nm. Chinese authors designed a system consisting of a light-emitting diode (LED), a pH-sensing and -monitoring module, and radio communication, as well as control and power management. The LEDs included in the capsule emitted blue light at 408 ± 2 nm with a radiation energy of 140 mW/cm^2^. A pH sensor measuring values from 1 to 9 was included to indicate the capsule location. A wireless communication module was employed to measure the pH values of the receiver. The capsule came into contact with the gastric wall and juice. The experimental test proved that the capsule emitted light for 32 min, but in vivo tests are lacking. The capsule was developed in 2016, and since then, no new data have been published [95].

## 7. Probiotics

Probiotics are a recurring topic in an attempt to improve the eradication of *H. pylori* infection. The use of probiotics as an additive treatment was proposed in 1995, and since then, several hundreds of microbiological and clinical studies have been performed. Probiotics have different mechanisms of action on *H. pylori*, as follows:

(a) The inhibition of bacterial adhesion to the surface epithelial cells;
(b) The inhibition of bacterial enzymes (urease, catalase, and carbonic anhydrase);
(c) The secretion of antibacterial substances (bacteriocins such as reuterin, lacticin, bulgaricin, etc.);
(d) The inhibition of biofilm formation;
(e) Host cell immune modulation (monitoring the balance of pro- and anti-inflammatory cytokines) [96].

The biologic relevance of all these mechanisms is largely disputed. The experimental data produced strong arguments for an anti-*H. pylori* action of some probiotics (*Lactobacilus acidophilus*, *Lactiplantibacillus pentopsus SLC13*, *Limosilactobacillus reuteri*, *Bifidobacterium species*, *Bacillus crusei*, and *Saccharomyces boulardii*). However, the clinical data are not so convincing. A recent meta-analysis of 11 studies, including 403 cases, showed that monotherapy with probiotics (*Lactobacilli* and *Saccharomyces boulardii*) obtained a mean weighted eradication rate of only 14% (95% CI: 2–25%) [97]. None of the bacteriocins secreted by probiotics was further developed as an antibiotic.

Between 2007 and 2019, 20 meta-analyses were performed, with contradictory results. Using A Measurement Tool to Assess Systematic Reviews (AMSTAR-2), it was shown that the quality of these studies was rather moderate, so the resulting evidence was also not very strong [98,99]. Consequently, the recommendations of the guidelines are controversial (Table 4) [4,5,6,7,8].

A recent high-quality meta-analysis based on 8924 patients from 40 studies stated that probiotics given with different eradication regimens achieved a rate of 81.5%, while controls only reached 71.6%, (*p* < 0.001, I^2^ = 52.1%). Probiotics used before and throughout eradication for periods longer than 2 weeks were more efficient than those given during or after treatment for a shorter period. The most efficient were bismuth-based quadruple regimens combined. Multi-strain preparations were better than other strains [100].

Most recently, Greek authors reported that a four-probiotic-containing preparation (*Lactobacillus acidophilus*, *Lactiplantibacillus plantarum*, *Bifidobacterium lactis*, and *Saccharomyces boulardii*) added to a 10-day non-bismuth quadruple treatment eradicated *H. pylori* in 92.0% of the patients, as compared to 86.8% of controls (*p* = 0.028), along with a significant reduction in side effects [101]. Conversely, monotherapy with *Limosilactobacillus reuteri* DSM 17648 improved the results of triple and quadruple regimens in many studies both in adults and children and reduced the side effects [102].

None of the current guidelines recommend the routine use of probiotics as an addition for eradication. The overwhelming diversity of the marketed preparations makes choosing a suitable preparation difficult for both the physician and the patient. In our practice, probiotics are offered to elderly patients with concomitant diseases, those prone to an altered microbiome, those with antecedent antibiotic-induced diarrhoea, or those with other side effects occurring with previous eradication regimens, as well as at the patient’s request.

## 8. Eradication of the Infection in Regions with a High *H. pylori* Prevalence

The prevalence of *H. pylori* infection is decreasing in developed countries, but it remains high, especially in the most populous regions. In recent decades, national, regional, and continental guidelines have been developed in many countries, which have both similarities and differences compared to the Western guidelines (4–8), trying to adapt the diagnostic and therapeutic approaches to the local conditions, drug availability and prices, and antimicrobial resistance profile.

The prevalence of the infection in China was 63.8% in 1983–1994, 57.5% in 1995–2005, and 46.7% in 2006–2018, declining by 0.9% annually, with high regional differences. Since 2017, four major consensus reports for *H. pylori* infection have been published [103]. A dose of A 2 × 1000 mg/day or 3–4 × 500 mg/day is recommended as a first antibiotic, and C 2 × 500 mg, LEV 1 × 500 mg, M 3–4 × 400 mg, or F 2 × 100 mg as a second antibiotic, along with 120 mg potassium bismuth citrate and a standard dose of PPI for 10–14 days. In a recent nationwide questionnaire survey conducted in 1000 hospitals, 88.0% of the respondents indicated adherence to the consensus statements, with considerable differences among gastroenterologists, general practitioners, professional titles, years of working, and regions, leaving scope for more education in the future [104].

In South Korea, with an infection prevalence of 50%, a PPI + A 2 × 1000 mg/day + C 2 × 500/day for 14 days or sequential/concomitant therapy for 10 days (PPI+A+C+M) are the proposed, first-line treatments, and bismuth-based therapy with T 4 × 500 mg/day and M 3 × 400 mg for 10–14 days is less preferred. Bismuth quadruple therapy is the second-line, and L-based triple or quadruple or rifabutin-based regimens are reserved for the second- or third-line treatments. All these regimens are based on evidence from controlled studies [105].

In Japan, the prevalence is 37.6–43.2%, and the recommended first-line treatment consists of PPI or VPZ + A 2 × 750 mg and C 2 × 200 mg for 7 days, followed by PPI + A 2 × 750 mg and M 2 × 250 mg for 7 days as second-line therapy. STF is added to the latter regimen at 2 × 100 mg/day as one of the drugs proposed to use in the third-line treatment [106]. VPZ is available only in Japan and South Korea. Bismuth compounds are not included in the most recent Japanese guideline [106].

The availability of antimicrobial resistance—especially genetic—tests varies greatly among these countries, and reimbursements from the national insurance systems are also different [107].

In India, the prevalence of the infection is around 80%. The 2021 Indian consensus recommended standard doses of PPI+A+C for 2 weeks and bismuth-based quadruple therapy in C-resistant areas. FQs are only allowed after two failed treatments, and nitroimidazoles are not recommended. While the resistance rates in India are high, culture is indicated only before third-line therapy and is not compulsory [108].

In Brazil, epidemiological studies have shown infection rates of 70% and 90% in urban and rural areas and children aged <10 years. Consensus meetings were held in 1995, 2004, 2012, and most recently, in 2018 [109]. Currently, standard PPI+A+C is the recommended first-line therapy. The alternatives are bismuth-based quadruple regimens containing T and M or A+C+M or tinidazole-containing concomitant regimens, with LEV or F being reserved for second- or third-line treatments.

Colombia has had its own national guideline since 2015. In this country, the prevalence of *H. pylori* is between 77 and 83%, and it is considered a public health problem. Data from the Colombian Health System cover 8.5 million people, out of which 12,011 patients with acid-peptic disease underwent 12,426 eradication treatments. Only 56.1% of the patients received adequate treatment, with 42% receiving a PPI+A+C, and 26.1% PPI+A+M. In spite of the guidelines, bismuth was given only in 0.2% of the patients. Reports from general practice showed that 20% of practitioners used a single antibiotic, and 2.6% did not use PPIs. The authors concluded that the management of *H. pylori* infection is heterogenous and inconsistent with the current recommendations [110]. This is similar to the conclusions drawn by the analysis of Hp-EuReg data based on 21,533 patients monitored for 5 years [11], leaving much room for education and improvement in the future [12].

## 9. Future Perspectives

The current therapeutic regimens against *H. pylori* are far from ideal, and there is a need for the identification and validation of new targets that result in more efficient drugs. Searching for innovative drugs involves research into the bacterium and host genomics, an investigation into gastric biofilms, targeting proteins involved in bacterial metabolism (purine nucleosidase phosphorylase, inosine-5′-monophosphate dehydrogenase, flavodoxin), and targeting virulence factors (CagA, outer membrane proteins, etc.). All these strategies may result in more efficient compounds. Bacteriocins produced by probiotics might also be developed into antibiotics. As the authors are clinical practitioners, this topic is far beyond their expertise [1,2].

## 10. Conclusions

The management of *H. pylori* infection entails many problems. In addition to the clinical setting of the patients, the infecting strains, nutrition and hygiene, the local profile of antibiotic resistance, and the availability of diagnostic methods and drugs can all influence eradication. Progressing from classical 7-day triple therapies to 10–14 days of quadruple, bismuth-based, or concomitant regimes is a slow process. P-CABs given as first- or second-line therapies are superior to PPI-based regimens. No innovative antimicrobials have been synthetised. Capsules containing antibiotics and bismuth— given with a PPI—have produced excellent results. The rational use of probiotic strains active against *H. pylori* could enhance the efficacy of its eradication.

## Figures and Tables

**Table 2:** Proton-pump inhibitors and potassium-competitive acid blockers: a comparison [1,2,24].

Pharmacologic Feature	PPI	P-CAB ^x^
Chemical structure	Substituted benzimidazoles	Revaprazan Vonoprazan Tegoprazan
Steady state after oral dosing	3–5 days	1 day
Plasma half-life	2 h	8–17 h
CYP2C19 polymorphism	Differential influence	No influence
Proton pump activation	Necessary	Not necessary
24 h intragastric pH > 4	46–58%	82.9–85.9%
Acid suppression at night % (pH > 4 HTR)	12.9 ± 10.9 (ESO), 15.3–13.3 (R)	67.9 ± 28.3.
Effect on *H. pylori*	Urease inhibition	Urease inhibition
Effect on urea breath test	Reduction of DOB‰, may result in a false negative test	Reduction of DOB‰, may result in a false negative test
Influence of meal	Reduced effect after meal	No influence
Short-term side effects	Headache, rush, dizziness, constipation, diarrhoea, flatulence, abdominal pain	Diarrhoea, constipation, eczema, upper respiratory tract inflammation in <5% of cases
Long-term side effects ^xx^	Fundic polyps, B_12_ vitamin and micronutrient deficiency, liver disease, hypomagnesemia, kidney disease	Under investigation

x: Some P-CABs were synthetised, but abandoned because of toxicity, while others are under investigation (DWP14012, YH4708, KFP-H008) in Japan, South Korea, and China. xx: Of no importance during the wo weeks of eradication; CYP: Cytochrome P450, DOB‰: delta over baseline ‰, ESO: esomeprazole, HTR: holding time ratio, P-CAB: potassium-competitive acid blocker, PPI: proton-pump inhibitor, R: rabeprazole.

**Table 3:** Meta-analyses comparing the effect of vonoprazan with PPI-based regimens.

Ref. No.	Year	Country	No. of Studies	No. of Patients	Regimens Used	Eradication Rates (ITT/PP), PPI-Based Regimens	Eradication Rates (ITT/PP), P-CAB-Based Regimens	Difference in Eradication Rates (ITT/PP)
[27]	2017	China	14	14,636	7 days of PPI- or VPZ-based triple regimens	68.0/74.2%	85.1/89.0%	+17.1/14.8%
[28]	2017	South Korea	10	10,644	PPI- or VPZ-based triple regimens	73.3/76.3%	88.1%/89.2%	+14.8/12.9%
[29]	2018	Japan	5	1599	PPI- or VPZ -based 7-day triple therapy in C-sens and C-res cases	C-sens: 92.8% C-res: 41.8%	C-sens: 95.8% C-res: 60.8%	C-sens: +3% C-res: +19%
[30]	2022	China	7	1168	PPI- or VPZ-based triple therapy	75.5/77.8%	90.2/93.0%	+14.6/14.2%

C-res: clarithromycin-resistant, C-sens: clarithromycin-sensitive, ITT: intention-to-treat analysis, P-CAB: potassium-competitive acid blocker, PP: per protocol analysis, PPI: proton-pump inhibitor, VPZ: vonoprazan.

**Table 4:** Probiotics and *H. pylori* eradication: guideline recommendations.

Ref. No.	Year	Guideline/Consensus	Recommendation	Evidence Level/Agreement	Grade
[4]	2016	Toronto	Not recommended	Low	C
[5]	2017	American College of Gastroenterology	Although probiotics are useful, the timing and optimal dosing (before/during/after treatment) are not decided	Not given	Not given
[6]	2016	Maastricht V/Florence	Certain probiotics may have beneficial effect on *H. pylori* eradication	Very low	Weak
[7]	2019	Reconciliation guideline	Despite current uncertainties, probiotics may still offer significant potential, and their influence on *H*. *pylori* eradication is worthy of further study	Not given	Not given
[8]	2022	Maastricht VI/Florence	Certain probiotics may have beneficial effects on *H. pylori* eradication therapy through reduction of antibiotic side effects	80%	B2

## Data Availability

No new data were created or analysed in this study. Data sharing is not applicable.
